# TonEBP/NFAT5 regulates ACTBL2 expression in biomechanically activated vascular smooth muscle cells

**DOI:** 10.3389/fphys.2014.00467

**Published:** 2014-12-03

**Authors:** Maren Hödebeck, Clemens Scherer, Andreas H. Wagner, Markus Hecker, Thomas Korff

**Affiliations:** Division of Cardiovascular Physiology, Institute of Physiology and Pathophysiology, University of HeidelbergHeidelberg, Germany

**Keywords:** smooth muscle cells, biomechanical stretch, hypertension, NFAT5, migration

## Abstract

Cytoskeletal reorganization and migration are critical responses which enable vascular smooth muscle cells (VSMCs) cells to evade, compensate, or adapt to alterations in biomechanical stress. An increase in wall stress or biomechanical stretch as it is elicited by arterial hypertension promotes their reorganization in the vessel wall which may lead to arterial stiffening and contractile dysfunction. This adaptive remodeling process is dependent on and driven by subtle phenotype changes including those controlling the cytoskeletal architecture and motility of VSMCs. Recently, it has been reported that the transcription factor nuclear factor of activated T-cells 5 (TonEBP/NFAT5) controls critical aspects of the VSMC phenotype and is activated by biomechanical stretch. We therefore hypothesized that NFAT5 controls the expression of gene products orchestrating cytoskeletal reorganization in stretch-stimulated VSMCs. Automated immunofluorescence and Western blot analyses revealed that biomechanical stretch enhances the expression and nuclear translocation of NFAT5 in VSMCs. Subsequent *in silico* analyses suggested that this transcription factor binds to the promotor region of ACTBL2 encoding kappa-actin which was shown to be abundantly expressed in VSMCs upon exposure to biomechanical stretch. Furthermore, ACTBL2 expression was inhibited in these cells upon siRNA-mediated knockdown of NFAT5. Kappa-actin appeared to be aligned with stress fibers under static culture conditions, dispersed in lamellipodia and supported VSMC migration as its knockdown diminishes lateral migration of these cells. In summary, our findings delineated biomechanical stretch as a determinant of NFAT5 expression and nuclear translocation controlling the expression of the cytoskeletal protein ACTBL2. This response may orchestrate the migratory activity of VSMCs and thus promote maladaptive rearrangement of the arterial vessel wall during hypertension.

## Introduction

The defined arrangement of vascular smooth muscle cells (VSMCs) and extracellular matrix in the media of the arterial wall provides the structural basis for the physiological properties of the arterial system. On the one hand, this flexible architectural concept allows for the development of elastic but less contractile conduit arteries or less elastic but highly contractile resistance arteries. On the other hand, pathophysiological alterations in the circulatory system may be compensated by adapting matrix structure and cellular arrangement in the arterial wall. Even a subtle but chronic increase in arterial pressure as it may occur during essential or idiopathic hypertension is known to cause a slowly progressive structural rearrangement of the arterial media leading to arterial stiffening and contractile dysfunction. Besides proliferation, repositioning and therefore migration of VSMCs is a prerequisite for this process.

During hypertension-induced arterial remodeling, a chronic increase in wall stress or biomechanical stretch is likely to act as the most important determinant promoting activation and migration of VSMCs within the media of the artery (Olivetti et al., 1982; Feldner et al., 2011; Pfisterer et al., 2012). Directed migration requires a polarized reorganization of the actin cytoskeleton to define the cell's front with a loose actin scaffold and the cell's rear with stabilized (stress) fibers. Depending on the local balance of stabilizing and destabilizing mechanisms, globular actin (G-actin) rapidly polymerizes to form filamentous actin (F-actin) or depolymerizes to liberate G-actin monomers. From the six mammalian actin genes, the regulation of cytoskeletal actins ACTB (β-actin) and ACTG2 (γ-actin) has been studied in some detail whereby the latter encodes for the largest actin isoform and its expression is regulated by CArG promotor elements as a target of the transcriptional coactivator myocardin (Sun et al., 2009). Moreover, the expression of the VSMC-specific α-smooth muscle actin (αSMA) appears to be controlled by both myocardin (Li et al., 2003) and the hypertonicity-responsive transcription factor nuclear factor of activated T-cells 5 (NFAT5) (Halterman et al., 2011).

NFAT5 is known to be involved in controlling the expression of genes involved in cellular homeostasis (Miyakawa et al., 1999), migration and proliferation of cells (Jauliac et al., 2002; Go et al., 2004; O'connor et al., 2007). In VSMCs, its expression and activity is regulated by platelet derived growth factor BB (PDGF-BB)—a humoral factor that drives mitogenic responses and chemotactic migration. In a recent study, we revealed that biomechanical stretch of VSMCs promotes expression and translocation of NFAT5 into the nucleus. As a consequence, NFAT5 up-regulates the expression of tenascin-C—a protein of the extracellular matrix that orchestrates the migration of VSMCs (Scherer et al., 2014). However, impaired migration of NFAT5-deficient cells was only partially rescued by exogenous TNC indicating that this transcription factor may control the expression of other genes rate-limiting for this cellular activity. While the general impact of NFAT5 on cellular migration has repeatedly been reported (O'connor et al., 2007; Halterman et al., 2012a), the molecular basis of corresponding context-specific functions of NFAT5 in individual cell types was not much elucidated so far. Against this background, our study investigated the impact of NFAT5 on the expression of novel cytoskeletal proteins which may contribute to the migratory capacity of VSMCs.

## Materials and methods

### Cell culture

Human arterial smooth muscle cells (HUASMCs) were freshly isolated from individual umbilical cords and grown on collagen I-bonded BioFlex® plates (Flexcell International, Hillsborough, NC, USA) with DMEM medium containing 15% FCS (osmolarity: 305 mosm ± 4.08 mOsm). The isolation of HUASMCs was approved by the local Ethics Committee (Heidelberg, Germany; reference 336/2005) and conformed to the principles outlined in the Declaration of Helsinki (1997). Stretch was typically applied at a frequency of 0.5 Hz and an elongation of 0–13% for 24 h by using a Flexercell FX-5000 tension system. In addition, cells were exposed to the following reagent: 40 μmol/L Etomoxir (Calbiochem, Germany). The compound was dissolved in DMSO. Pure DMSO (0.4%, v/v) was simultaneously applied to the control cells as solvent control.

### Immunocytochemistry

Cells were fixed in ice-cold methanol for 15 min and allowed to dry for 20 min. Rehydrated cells were blocked with 0.25% casein and 0.1% BSA for 30 min. Cells were incubated with rabbit anti-NFAT5 antibody 1:100 (sc-13035, Santa Cruz, CA) or anti-ACTBL2 antibody 1:100 (abcam Cambridge, UK) at 4°C overnight. After washing, cells were incubated with donkey anti-rabbit-Cy3 1:100 (Dianova, Hamburg, Germany) for 1 h and mounted with Mowiol (Calbiochem, Hilden, Germany). Nuclei were visualized by counterstaining the cells with DAPI (Invitrogen). Fluorescence intensity was recorded using a fluorescence microscope and quantitated utilizing either the Olympus Xcellence software or the TissueQuest Analysis software.

### Transfection with siRNA

HUASMCs were transfected with short interfering RNA directed against NFAT5 (5 ′-*CCA GTT CCT ACA ATG ATA A*-3′). As control commercially available siGENOME non-targeting siRNA (Thermo Scientific, Bonn, Germany) was applied. For each well of a 6-well plate, 3 μg of siRNA was diluted in Opti-MEM I (Invitrogen) together with 3 μl of MATra-si reagent (IBA, Göttingen, Germany) to give a final volume of 200 μl. After mixing and incubating for 20 min at ambient temperature, the solution was added onto the cells which had been cultured in 2 ml Opti-MEM I prior to the transfection. Cells were then incubated on a magnet plate (IBA) at 37°C and 5% CO_2_. After 15 min cells were washed and cultured in normal cell medium for a resting period of 48 h for NFAT5 knockdown.

### Analysis of gene expression

Total RNA was isolated from the cultured HUASMCs using the peqGOLD Total RNA Kit (Peqlab) according to the manufacturer's instructions. Subsequently, reverse transcription (RT) and quantitative polymerase chain reaction (qPCR) for ACTBL2 and 60S ribosomal protein L32 (RPL32) cDNA as an internal standard was performed. ACTBL2 primers were bought from Qiagen and primers for RPL32 were used for amplification based on the following sequence: *for 5* ′*-GTT CAT CCG GCA CCA GTC AG-3* ′*, rev 5* ′*-ACG TGC ACA TGA GCT GCC TAC-3* '

### Western blot

HUASMCs were lysed using sample buffer containing 1% Triton X-100 and 0.1 μmol/L DTT or buffers for preparing nuclear and cytosolic fractions. Protein samples were separated by SDS (10%), blotted onto nitrocellulose membranes and analyzed by chemiluminescence-based immunodetection according to standard procedures. Primary antibodies: rabbit anti-NFAT5 1:500 (Santa Cruz Biotechnology), rabbit anti-ACTBL2 (κ-actin) 1:1000 (abcam), mouse anti-**β**-actin 1:10,000 (abcam), rabbit anti-histone H3 1:1000 (abcam), rabbit anti-alpha-Tubulin (Cell Signaling) 1:1000.

### Nuclear extraction

Nuclear protein extraction was performed according to the following protocol: HUASMCs were lysed using buffer I containing 10 mM HEPES, 10 mM KCl, 1 μM EDTA, 1 μM EGTA, 15% Nonidet, protease and phosphatase inhibitors. After centrifugation (12,000 × g at 4°C for 15 min) the supernatant (cytosolic fraction) was transferred to a new tube and stored or immediately used for Western blotting. The remaining pellet containing the nuclear fraction was dissolved in 40 μl buffer II consisting of 20 mM HEPES, 400 mM NaCl, 0.01 M EDTA, 0.01 M EGTA, 15% Nonidet and protease and phosphatase inhibitors. Subsequently, this solution was sonicated two times for 5 s at 50 Watts at 4°C. After centrifugation (12,000× g at 4°C for 15 min) the supernatant containing the nuclear fraction was transferred to a new tube and stored at −80°C or was immediately used.

### Bioinformatic analysis

About 3203 bp of the promoter sequence upstream of the human ACTBL2 translation start site (Homo sapiens ACTBL2, accession NG_029637, position 28727 to 32239) were analyzed using the MatInspector software (Genomatrix Software, Munich Germany) for potential NFAT5 consensus sequences.

### Cell migration assay

Cultured HUASMCs were seeded into 24-well culture plates with 4 mm thick and 10 mm long silicon walls in the center of each well, after transfection with specific siRNA, control siRNA or left untreated. After 24 h the silicon walls were removed and after 48 h, the distance between the cell borders was measured at 2× magnification or cells were fixed and stained for κ-actin.

### Statistical analysis

All results are expressed as means ± *SD* of *n* individual experiments. Differences between individual experimental groups were analyzed by unpaired Student's *t*-test or non-parametric Mann–Whitney *U*-test, with *p* < 0.05 considered statistically significant.

## Results

### Biomechanical deformation of VSMCs promotes nuclear translocation of NFAT5

**Figure 1:**
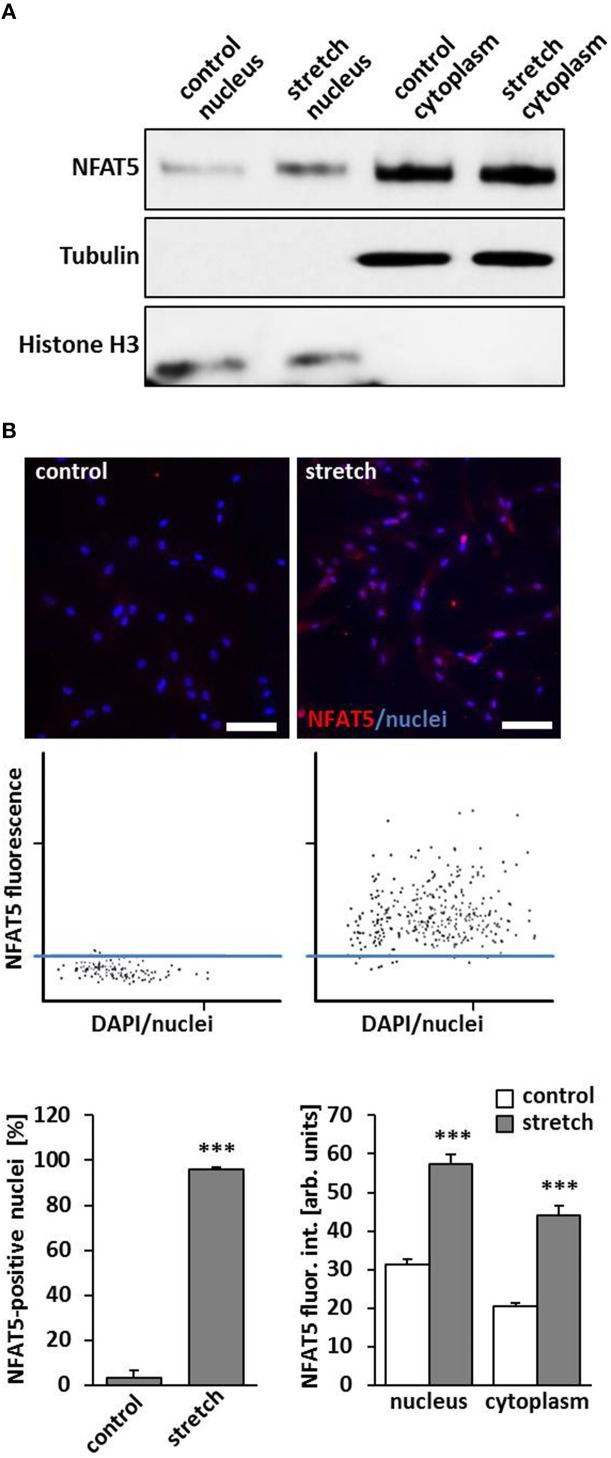
**Biomechanical stretch induces nuclear translocation of Nuclear factor of activated T cells 5 (NFAT5)**. HUASMCs were exposed to biomechanical stretch for 24 h. Subsequent Western blot analyses of nuclear and cytosolic protein fractions showed an increase of nuclear NFAT5 abundance compared to control conditions **(A)**. Automated immunofluorescence analyses (TissueGnostics/TissueQuest) revealed a significant increase of NFAT5 positive nuclei (**B**, ^***^p < 0.001 vs. control, *n* = 3) and NFAT5-specific nuclear/cytoplasmic fluorescence intensity (**B**, ^***^p < 0.001 vs. control, *n* = 3) in stretch-stimulated VSMCs which is also demonstrated by corresponding scattergramms. The threshold was set according to the basal NFAT5 fluorescence intensity under control conditions (scale bar: 100 μm).

A general response of VSMC on a biomechanical stimulation is an adaptive rearrangement of the cytoskeleton. This may either allow the cells to generate contractile forces acting against the stimulus or to evade them through local repositioning or migration, respectively. Corresponding changes in gene expression have to support any of these options and are controlled by concerted transcriptional activity. In this context, it has been shown that the transcription factor NFAT5 controls gene expression mediating activity and migration of VSMCs (Halterman et al., 2011) and is activated by biomechanical stimuli (Scherer et al., 2014). To verify that the transcription factor NFAT5 enters the nucleus under corresponding conditions, nuclear and cytoplasmic protein extracts were analyzed and revealed an increased level of NFAT5 in the nuclei of stretch-stimulated VSMCs (Figure 1A). Likewise, automated tissue-FACS-based immunofluorescence analyses indicated an elevated NFAT5-specific fluorescence intensity in the nuclei and cytoplasm in biomechanically stimulated VSMCs (Figure 1B). To exclude any impact of the osmolarity of the culture medium on NFAT5 activity under the chosen experimental conditions, this parameter was determined upon exposing VSMCs to biomechanical stretch for 24 h but did not show any relevant changes (control: 305 ± 4.08 mOsm; stretch: 297 ± 2.89 mOsm, *n* = 4).

### NFAT5 controls expression and protein abundance of κ-actin in stretch-exposed VSMCs

**Figure 2:**
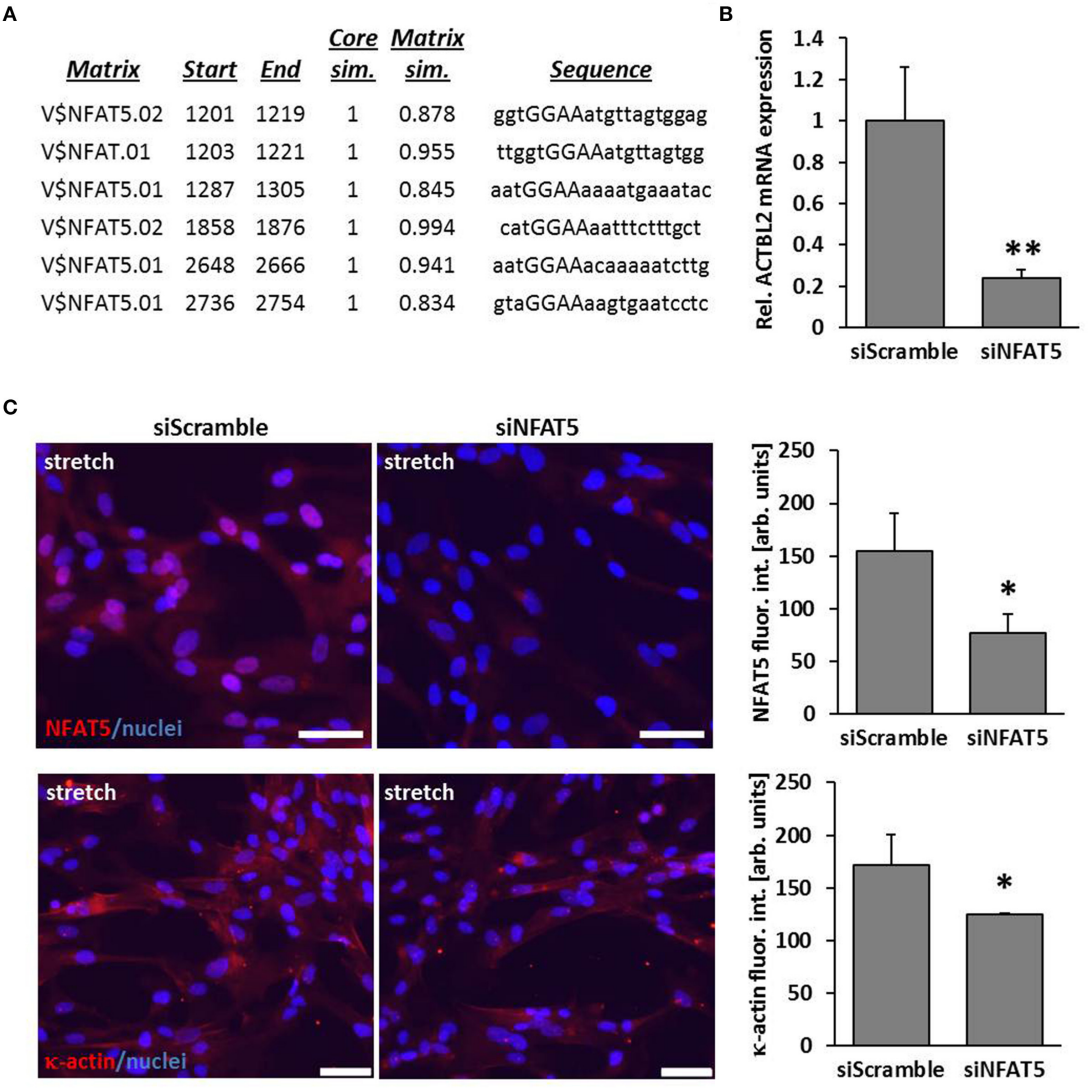
**ACTBL2 is a transcriptional target of NFAT5**. *In silico* promoter analysis of the human ACTBL2 promoter revealed six putative NFAT5 binding sites **(A)**. The first 3203 bp of the promoter sequence upstream of the transcription start site were analyzed. The maximum core similarity (Core sim.) of 1.0 is only reached when the highest conserved bases of a matrix are exactly matched by the sequence (cf. capitals in the sequence). A good match to the matrix has a similarity of >0.80 (Matrix sim.). Quantitative PCR (qPCR) of stretch-stimulated HUASMCs treated with NFAT5-specific siRNA (siNFAT5) showed a significant decrease in ACTBL2 mRNA expression compared to stretch-stimulated control siRNA(siScramble)-transfected cells (**B**, ^**^p < 0.01 vs. control siRNA, *n* = 3 scale bar: 20 μm). SiRNA-mediated knockdown of NFAT5 (**C**, ^*^p < 0.05 vs. siScramble) decreased κ-actin (**C**, ^*^p < 0.05 vs. siScramble) in stretch-stimulated VSMCs as evidenced by immunofluorescence analyses (scale bar: 100 μm).

By performing *in silico* analyses, we showed that the promotor sequence of the human ACTBL2 gene—enconding κ-actin, a novel member of the actin family that was originally detected in hepatoma cells (Chang et al., 2006)—contains several putative NFAT5 binding sites (Figure 2A). Consequently, knockdown of NFAT5 by siRNA induced a significant reduction in expression of the ACTBL2 gene (Figure 2B) and decreased the abundance of NFAT5 as well as κ-actin in the nuclei or cyotoplasm of stretch-stimulated VSMCs (Figure 2C).

### Expression of ACTBL2 in stretch-exposed VSMCs is dependent on palmitoylation of NFAT5

**Figure 3:**
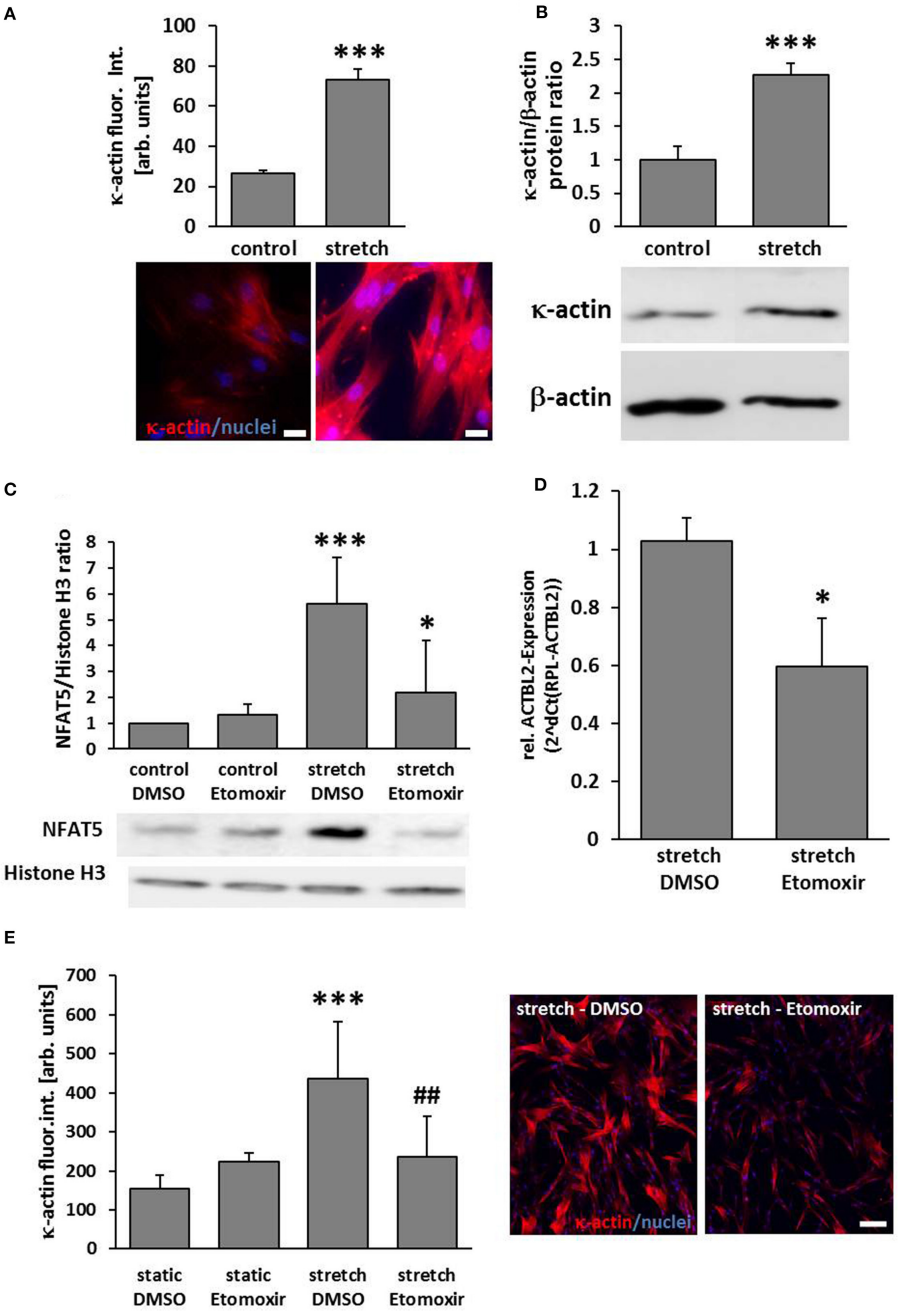
**ACTBL2 expression in stretch-stimulated SMCs is dependent on the Carnitine palmitoyltransferase family 1 (CPT1)**. Immunofluorescence analyses indicated a significant increase of κ-actin abundance in HUASMCs upon stretch stimulation (**A**, ^***^p < 0.001 vs. control, *n* = 3; scale bar: 20 μm) which was confirmed by Western blot analyses (**B**, ^***^p < 0.001 vs. control, *n* = 3). Etomoxir, a specific CPT1-inhibitor (40 μM) reduced the stretch-induced NFAT5 translocation which was determined by analyzing nuclear protein extracts (**C**, ^***^p < 0.001 control DMSO vs. stretch DMSO, ^*^p < 0.05 stretch DMSO vs. stretch Etomoxir, *n* = 5). Quantitative real-time PCR revealed a decline in ACTBL2 mRNA expression in stretch-stimulated Etomoxir-treated HUASMCs (**D**, ^*^p < 0.05 vs. stretch DMSO, *n* = 3). Stretch-dependent (24 h) increase in κ-actin protein abundance is attenuated upon treatment with Etomoxir as evidenced by immunofluorescence detection (**E**, ^***^p < 0.001 vs. static DMSO, ^##^*p* < 0.01 vs. stretch DMSO; bar graphs represent the mean (±*SD*) fluorescence intensity of κ-actin in ten microscopic fields of view; representative images are shown on the right, scale bar: 100 μm).

According to the aforementioned findings, the abundance of κ-actin was significantly increased in biomechanically stimulated VSMCs (Figures 3A,B). As earlier results suggested that NFAT5 activity is regulated by stretch-dependent palmitoylation (Eisenhaber et al., 2011; Scherer et al., 2014) we assumed that the class of carnitine palmitoyl transferases is crucial for the palmitoylation process and thus NFAT5 activity and κ-actin expression. We scrutinized this hypothesis by investigating stretch-induced nuclear translocation of NFAT5 in VSMCs which were treated with etomoxir—an inhibitor of the mitochondrial carnitine palmitoyltransferase 1 that is tested in clinical trials for its capacity to treat congestive heart failure (Holubarsch et al., 2007). While this drug inhibited NFAT5 from entering the nucleus (Figure 3C), it also attenuated the gene expression of the ACTBL2 (Figure 3D) and protein expression (Figure 3E).

### κ-actin is involved in stress fiber organization and migration of VSMCs

**Figure 4:**
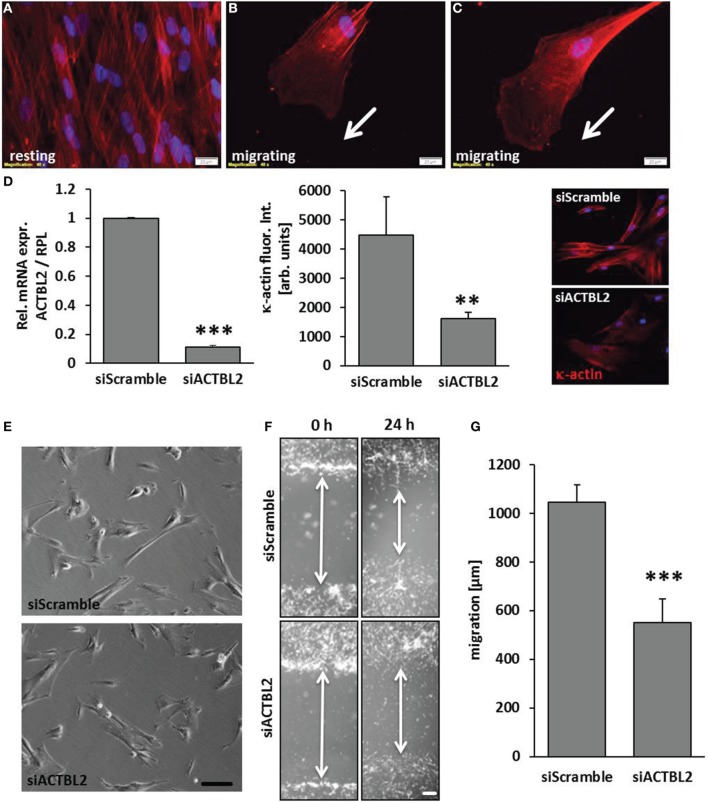
**κ-actin modulated VSMC migration**. κ-actin is localized in stress fibers of resting cells **(A)** and dispersed in protrusions of migrating cells (**B,C**) as evidenced by contrast enhancing immunofluorescence detection. ACTBL2 knockdown efficiency was verified by RT-PCR and immunofluorescence analysis 2 days after transfection (**D**, ^**^p < 0.01 and ^***^p < 0.001 vs. siScramble, *n* = 3) and did not affect spreading or viability of the HUASMCs (**E**, scale bar: 100 μm). In contrast to control, planar cell migration is inhibited in HUASMCs after ACTBL2 silencing (**F,G**, ^***^p < 0.001 vs. siScramble, *n* = 6, scale bar: 50 μm).

Orchestrated and continuous architectural adaptation of the cytoskeleton is a prerequisite for repositioning or migration of cells and defines a typical response to an altered biomechanical load. Structurally, the β-actins constitute the most relevant and abundant proteins of the cytoskeleton and are thus rate-limiting for its adequate function. However, the function of κ-actin in this context has not been explored so far. High resolution contrast enhancing immunofluorescence analyses showed that this protein is located along stress fibers in resting VSMCs (Figure 4A). Alike β-actin, dispersed κ-actin accumulates at the border of the lamellipodia, is basically absent from the migration front and implemented in stress fibers in the cell's rear (Figures 4B,C). As silencing of ACTBL2 gene expression (Figure 4D) had no effect on spreading or morphology of resting cells (Figure 4E), we next analyzed its impact on the dynamic rearrangement of the cytoskeleton as it usually occurs during cellular migration. By utilizing a lateral sheet migration assay, we revealed that knockdown of κ-actin impairs VSMCs migration (Figures 4F,G). Furthermore, detailed time-lapse analyses of these cells indicated that silencing of ACTBL2 expression partially impairs directional migration (Supplementary Videos 1, 2) and thus led to a 3- to 4-fold increase in basically immobile cells.

## Discussion

NFAT5 was originally identified as a hypertonicity-responsive transcription factor that regulates cellular homeostasis (Miyakawa et al., 1999). By triggering phosphorylation of the carboxy-terminal transactivation domain of NFAT5, osmotic stress promotes its nuclear translocation and transcriptional activity (Lopez-Rodriguez et al., 1999; Miyakawa et al., 1999; Dahl et al., 2001; Ferraris et al., 2002; Lee et al., 2003). Meanwhile, NFAT5 has also been reported to control gene expression in angiotensin II- or PDGF-BB-stimulated VSMCs (Halterman et al., 2011). Thereby, it contributes to a phenotypic switch of these cells which includes the transition from a quiescent and resting to an activated and motile phenotype that is usually associated with vascular remodeling processes. An important determinant mediating such a phenotype change and realignment of VSMCs in the arterial media is a chronic increase in wall stress or biomechanical stretch. Elevation of these forces occurs during hypertension (Olivetti et al., 1982; Haudenschild et al., 1985) which is accompanied by thickening, stiffening and malfunction of the arterial vessel wall (Olivetti et al., 1982; Intengan and Schiffrin, 2000). Changes in cellular activity preceding these structural alterations are controlled by a wide range of transcription factors such as AP-1 and SRF (Wang et al., 2004; Demicheva et al., 2008). In this context, we revealed that stretch-stimulated VSMCs respond by enhancing protein abundance and nuclear translocation of NFAT5 (Scherer et al., 2014) which is in line with observations made in this study. Although several kinases have been reported to control NFAT5 activity (Jauliac et al., 2002; Halterman et al., 2012b), p38 MAP kinase and ERK1/2-dependent signaling appear to affect neither expression nor translocation of NFAT5 under these conditions. However, its palmitoylation appears to be crucial for the stretch-induced nuclear translocation—a covalent attachment of fatty acids to cysteine, sometimes serine and threonine residues of the protein core. Such a modification has been reported to regulate the entry of NFAT5 into the nucleus in response to osmotic stress (Eisenhaber et al., 2011). Additionally, our study suggests that the activity of type 1 carnitine palmitoyl transferases (CPT1) is rate-limiting for the translocation process occurring in VSMCs upon exposure to biomechanical stretch.

Impaired nuclear translocation as well as knockdown of NFAT5 attenuated the expression of its transcriptional target genes such as tenascin-C which has been identified in earlier studies. With ACTBL2–a gene encoding κ-actin–we revealed a novel NFAT5 target whose product constitutes one component of the VSMC cytoskeleton. κ-actin is a 42 kDa actin isoform that was originally detected in hepatoma cells (Chang et al., 2006) and showed single nucleotide polymorphisms in its promotor that were associated with orthostatic hypotension and supine-standing blood pressure changes (Hong et al., 2013). At first glance, κ-actin behaves like β-actin as it is organized in stress fibers and follows a distribution in migrating cells as has been repeatedly described for β actin (Gerthoffer, 2007). For directed migration, cellular lamellipodia are extended by actin polymerization and nucleation of new filaments which is governed by a plethora of actin-binding proteins such as the actin-related protein 2/3 (ARP2/3) complex. However, phylogenetic analyses indicated that the ACTBL2 sequences were genetically distant from those encoding α-, β-, and γ-actin (Chang et al., 2011). Thus, κ-actin may interact with an individual set of proteins. For instance, interaction of κ-actin and prefoldin 2 but not 1—members of a heterohexameric chaperone protein family that is capable to capture unfolded actin (Vainberg et al., 1998)—appear to be diminished as compared to β-actin (Chang et al., 2006). On the functional level, increasing the protein abundance of κ-actin in stretch-stimulated VSMCs must have an advantage for the cells as may be deduced from their impaired migratory capacity upon silencing ACTBL2 gene expression. In fact, increased levels of κ-actin expression are preserved in the course of hepatocarcinogenesis and grants hepatoma cells a growth advantage. Likewise, abundant κ-actin levels are associated with a poorer postoperative disease-free survival of hepatocellular carcinoma patients (Chang et al., 2011) which may also be based on an enhanced migratory capacity of these cells. Therefore, it is tempting to speculate that etomoxir may diminish metastasis of distinct tumors by indirectly inhibiting expression of κ-actin and the activity of the pro-migratory transcription factor NFAT5.

Collectively, our findings suggest that (i) biomechanical stretch promotes the CPT1-dependent nuclear translocation of NFAT5 into the nucleus of cultured human arterial smooth muscle cells and (ii) this transcription factor controls the expression of κ-actin that contributes to VSMC migration. In the context of hypertension these cascade of events may support VSMCs in acquiring an activated phenotype. The stretch-induced translocation of NFAT5 therefore constitutes a novel regulatory mechanism underlying this phenomenon during stretch or wall stress-induced maladaptive remodeling processes which occur in the early phases of hypertension or atherosclerosis.

## Funding

This work was supported by a grant from the Deutsche Forschungsgemeinschaft (SFB TR 23, project sections C5 and C6).

### Conflict of interest statement

The authors declare that the research was conducted in the absence of any commercial or financial relationships that could be construed as a potential conflict of interest.
