# Supplementary material for: TonEBP/NFAT5 regulates ACTBL2 expression in biomechanically activated vascular smooth muscle cells
Source: Front Physiol. 2014 Dec 3;5:467. doi: 10.3389/fphys.2014.00467 (PMC4253659; doi:10.3389/fphys.2014.00467)
Supplement: Supplementary file 3 [file Presentation1.PDF]

MOVIES:

113437_Korff_Video 1.MP4:
Directed migration of control-treated VSMCs (siScramble) forming normal lamellipodia.

113437_Korff_Video 2.MP4:
Disturbed (ineffective) migration of siACTBL2-treated VSMCs (siScramble) with disordered formation of lamellipodia.
